# *C3H* Expression Is Crucial for Methyl Jasmonate Induction of Chicoric Acid Production by *Echinacea purpurea* (L.) Moench Cell Suspension Cultures

**DOI:** 10.3390/ijms231911179

**Published:** 2022-09-23

**Authors:** Laura Ravazzolo, Benedetto Ruperti, Marco Frigo, Oriana Bertaiola, Giovanna Pressi, Mario Malagoli, Silvia Quaggiotti

**Affiliations:** 1 Department of Agronomy, Food, Natural Resources, Animals and Environment, University of Padova, Viale dell’Università 16, 35020 Legnaro, PD, Italy; 2 Aethera Biotech srl, Via dell’Innovazione 1, 36043 Camisano Vicentino, VI, Italy

**Keywords:** *Echinacea purpurea*, chicoric acid, plant cell culture, gene expression, methyl jasmonate

## Abstract

*Echinacea purpurea* (L.) Moench is one of the most economically important medicinal plants, cultivated worldwide for its high medicinal value and with several industrial applications in both pharmaceutical and food industries. Thanks to its various phytochemical contents, including caffeic acid derivatives (CADs), *E. purpurea* extracts have antioxidant, anti-inflammatory, and immuno-stimulating properties. Among CADs, chicoric acid is one of the most important compounds which have shown important pharmacological properties. The present research was aimed at optimizing the production of chicoric acid in *E. purpurea* cell culture. Methyl jasmonate (MeJa) at different concentrations and for different duration of treatments was utilized as elicitor, and the content of total polyphenols and chicoric acid was measured. Several genes involved in the chicoric acid biosynthetic pathway were selected, and their expression evaluated at different time points of cell culture growth. This was performed with the aim of identifying the most suitable putative molecular markers to be used as a proxy for the early prediction of chicoric acid contents, without the need of expensive quantification methods. A correlation between the production of chicoric acid in response to MeJa and an increased response to oxidative stress was also proposed.

## 1. Introduction

*Echinacea purpurea* (L.) Moench belongs to the *Echinacea* genus in the Asteraceae family as one of the most economically important medicinal plants [1,2,3]. Originally discovered in North America, *Echinacea* plants are now cultivated worldwide for their high medicinal value and several industrial applications in both pharmaceutical and food industries [4]. Thanks to their content of various phytochemicals, including caffeic acid derivatives (CADs), alkylamides and polysaccharides, *E. purpurea* extracts have antioxidant, anti-inflammatory, and immuno-stimulating properties [5,6,7]. They are used in the treatment of common cold and upper respiratory infections, by reducing duration and/or severity of symptoms. Among CADs, chicoric acid is one of the most important compounds which have shown important pharmacological properties, with *E. purpurea* showing the highest content of chicoric acid in the ethanolic extracts of flowers and roots [8]. For instance, treating leukemic mice for 50 days with *E. purpurea* extracts enhanced immune status and prolonged life span [9]. *E. purpurea*-containing products also stimulated the first defense against human influenza viruses by modulating their entry, internalization and replication [10].

Free radical-scavenging and antioxidant activities of *E. purpurea* extracts have been ascribed to phenolic constituents, including chicoric acid [11], resulting in mitigation of oxidative stress through neutralization of ROS generation, inhibition of lipid peroxidation, and glutathione adjustment [6].

Moreover, chicoric acid inhibits HIV-1 integrase and replication [12] and it exhibits potent antiviral effects against herpes simplex, influenza, vaccinia virus, vesicular stomatitis virus (VSV)-Ebola [13], and also against the SARS-CoV-2 infection [14].

As a CAD, chicoric acid is biosynthetically formed through the phenylpropanoid pathway, with phenylalanine as the precursor molecule [15,16]. Phenylalanine is then converted by phenylalanine ammonia-lyase (PAL) to cinnamic acid, which in turn is converted into p-coumaric acid by cinnamate 4-hydroxylase (C4H). P-coumaric acid is then converted into caffeic acid by the p-coumarate-3-hydroxylase (C3H), then the 4-(hydroxyl) cinnamoyl CoA ligase (4CL) catalyzes the conversion of p-coumaric acid and caffeic acid into p-coumaroyl CoA and caffeoyl CoA, respectively [17]. The final steps of the biosynthetic pathway of chicoric acid have been recently elucidated and involve three acyltransferases, namely hydroxycinnamoyl-CoA: tartaric acid hydroxycinnamoyl transferase (HTT), hydroxycinnamoyl-CoA: quinate hydroxycinnamoyl transferase (HQT), and chicoric acid synthase (CAS) [18]. HTT catalyzes caftaric acid biosynthesis from caffeoyl CoA, while HQT generates chlorogenic acid. Then, caftaric acid and chlorogenic acid are transferred from the cytosol into the vacuole, and chicoric acid is synthesized via the CAS.

Currently, the consumption of *Echinacea* extracts and whole plant products increased significantly in Europe and North America, becoming one of the largest sectors of the several billion-dollar herbal medicine market [16]. Nevertheless, the traditional cultivation of *Echinacea* is strongly affected by environmental stresses and growth conditions which could impact on the quality and quantity of the botanical materials obtained. [19]. To overcome these limitations, various in vitro methods have been developed for *Echinacea* species, particularly for *E. purpurea*, starting from a plant fragment to bioreactor cultures to obtain a defined and standardized phytocomplex titrated in chicoric acid. Bioreactors allow for the development of biomass with a higher concentration of bioactive compounds, ultimately making a more efficient industrial process [20,21]. In cell culture systems, the application of elicitors is one of the most effective methods to enhance the production of secondary metabolites. For instance, it was shown that a treatment with the phytohormone methyl jasmonate (MeJa) induced accumulation of phenolics in *E. purpurea* cell cultures [22]. Elicitors may act at different check points of the production of secondary metabolites, such as by triggering the expression of genes involved in the biosynthetic pathway [23].

Despite past advances in related disciplines allowed to deeply study model plants at the genomic, transcriptomic, and proteomic level, for non-model plants such as *E. purpurea* no reference genomes are available. However, some studies which employed RNA-sequencing (RNA-seq) techniques can provided large-scale transcriptomic information, thus allowing us to compare gene expression analysis in the biosynthesis pathways of chicoric acid of cell cultures differently elicited [4,24].

The present research was focused on optimizing the production of caffeic acid derivatives, in particular chicoric acid, in *E. purpurea cell* cultures. To this aim, the effect of different concentrations and duration of treatments with MeJa as elicitor was evaluated and the more suitable elicitation protocol was identified. Meanwhile, some genes putatively involved in chicoric acid biosynthesis pathway were selected, and their expression evaluated at different time points of cell culture growth with the aim of identifying putative molecular markers of chicoric acid biosynthesis to be used as a proxy for its production.

## 2. Results

### 2.1. Evaluation of the Methyl Jasmonate (MeJa) Optimum Concentration to Stimulate Chicoric Acid Biosynthesis in Echinacea Purpurea Cell Suspensions

**Figure 1:**
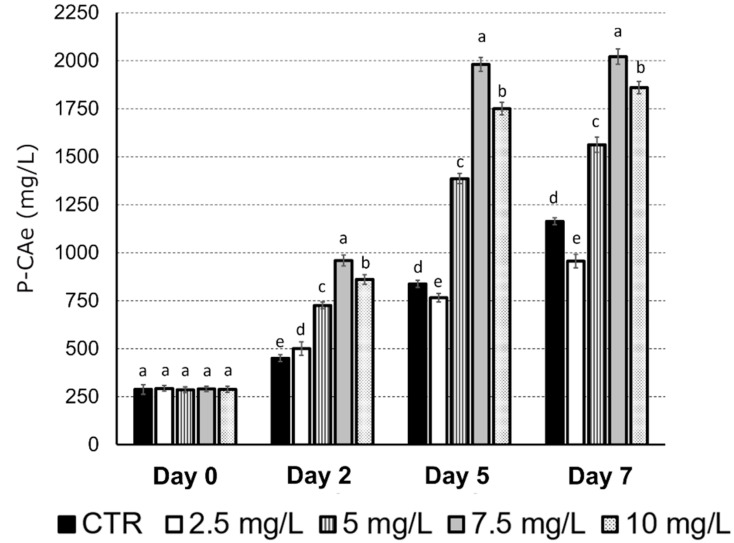
Content of total polyphenols, expressed as chicoric acid equivalent (P-CAe) after the addition of methyl jasmonate (MeJa) at different concentrations (2.5, 5, 7.5 and 10 mg/L) for 0, 2, 5 or 7 days. Data are mean ± SE for three biological replicates. Different letters above the bars indicate statistically significant differences at *p* < 0.05 for ANOVA.

Optimum methyl jasmonate (MeJa) concentration was selected according to total polyphenol content, expressed as a chicoric acid equivalent (P-CAe) of the *E. purpurea* suspensions culture after testing the addition of 2.5, 5, 7.5 and 10 mg/L of MeJa on the seventh day of growth in the EP-medium (Figure 1). After the addition of different concentrations of MeJa (day 0), the 4 treatments did not stimulate P-CAe accumulation if compared to the control (300 mg/L) (CTR, no MeJa added). After 2 days of treatment, the MeJa concentration of 7.5 mg/L induced the highest production of P-CAe compared to the CTR (+113%), followed by the treatment with MeJa 10 mg/L (+91%), MeJa 5 mg/L (+61%), and MeJa 2.5 mg/L (+11%). This trend was maintained during the following days, always with the treatment with MeJa 7.5 mg/L inducing the highest rate of P-CAe at all time-points, and in particular after 7 days (2021 mg/L, +74% with respect to the CTR). An exception was the treatment with 2.5 mg/L of MeJa that at 5 and 7 days induced a slight but significant reduction in the P-CAe content if compared to the control at the same time point (−9% and −18%, respectively). Furthermore, the P-CAe content constantly increased in the control without MeJa treatment during the seven days of cell culture growth, reaching after 7 days a 3-times higher content if compared to the starting point (1163 mg/L). Nevertheless, at this same time point (7 days) the provision of 7.5 mg/L of MeJa almost doubled the chicoric acid content. For this reason, 7.5 mg/L was selected as the optimum concentration to elicit the P-CAe production.

### 2.2. Total Polyphenols and Chicoric Acid Content in Echinacea Purpurea Cell Suspensions

**Figure 2:**
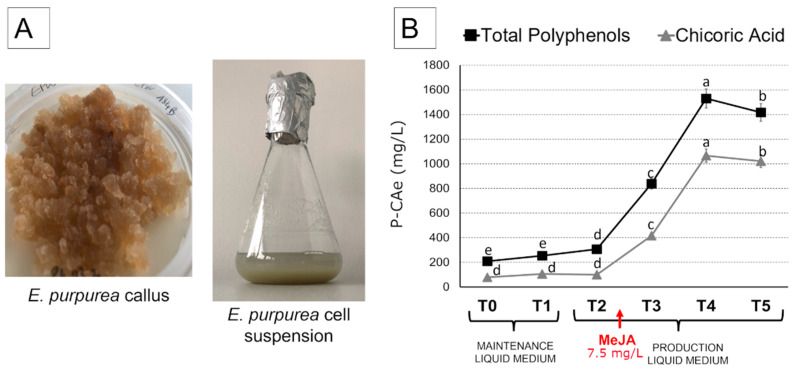
**Panel** (**A**): *E. purpurea* callus and cell suspension phenotype. **Panel** (**B**): Quantitative UPLC-DAD analysis results on total polyphenols expressed as chicoric acid equivalent (P-CAe) in *E. purpurea* cell suspensions at each time-point. Three biological replicates were analysed for each sample. Different letters above the bars indicate statistically significant differences at *p* < 0.05 for ANOVA.

*E. purpurea* calli were obtained from shoots and, after 6 months of subculture, they became friable and homogeneous, with a constant growth rate (Figure 2A). From selected calli, the suspension cultures were obtained and incubated for 7 days in the maintenance liquid medium before subculturing (T0 and T1). Then, they were transferred to the final productive liquid medium to induce the biosynthesis of chicoric acid and other polyphenols (T2, T3, T4, T5).

The content of P-CAe and chicoric acid contents (mg/L) in *E. purpurea* cell suspensions was assessed to evaluate their trend during time both in maintenance and in production liquid media (Figure 2B). In the maintenance medium (T0-T1) and also after 7 days in the productive liquid medium, but before the addition of the elicitor MeJa as 7.5 mg/L (T2), the chicoric acid content showed a constant level around 100 mg/L, with no significant variation among the three time points. Contrarily, P-CAe levels displayed a slight but significant increase between T1 and T2, reaching 307 mg/L (+47% if compared to T0). From T3, both chicoric acid and P-CAe showed a rapid increase, reaching a peak at T4 (1066 mg/L and 1530 mg/L, respectively) and slowly decreased in T5 (−4% and −7% with respect to T4, respectively) (Figure 2B).

### 2.3. Identification of Gene Sequences Involved in Chicoric Acid Biosynthesis in Echinacea Purpurea and Expression Analysis on Cell Suspensions to Select Markers Elicited by Methyl Jasmonate (MeJa)

**Figure 3:**
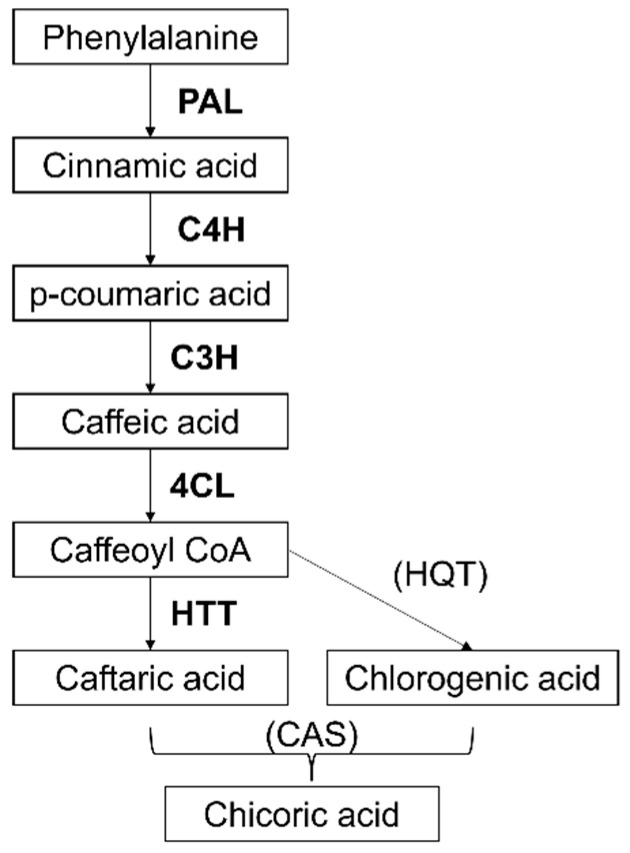
Simplified biosynthetic pathway of chicoric acid. The enzymes involved in each step are reported with their acronym in bold and their encoding genes were analyzed in this study, with the only exception of HQT and CAS (between bracket). Abbr: PAL—phenylalanine ammonia-lyase; C4H—cinnamate 4-hydroxylase; C3H—p-coumarate-3-hydroxylase; 4CL—4-(hydroxyl) cinnamoyl CoA ligase; HTT—hydroxycinnamoyl-CoA: tartaric acid hydroxycinnamoyl transferase; HQT—hydroxycinnamoyl-CoA: quinate hydroxycinnamoyl transferase; CAS—chicoric acid synthase. For more detailed information see [7,16].

The unigenes annotated by Tahmasebi and colleagues [24] were screened to find genes related to chicoric acid biosynthesis (Figure 3, Table S1). Before analyzing the expression in cell culture, genes related to chicoric acid biosynthesis were initially tested on samples obtained from *E. purpurea* tissues, in particular central florets, ligulate florets and young leaf, confirming their expression in vivo (Figure S1).

**Figure 4:**
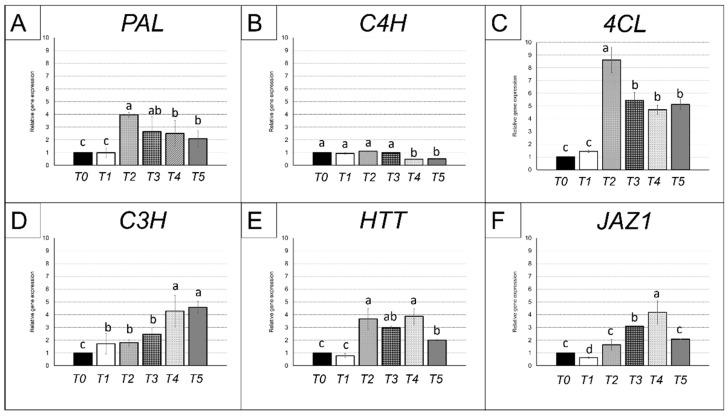
Expression analysis of genes involved in chicoric acid biosynthesis (**A**–**E**) and methyl-jasmonate signaling (**F**) on *Echinacea purpurea* cell suspensions, firstly grown in the maintenance liquid medium (T0-T1) and then in the productive liquid medium (T2-T3-T4-T5) for 21 days. After T2, MeJa was supplied to elicit chicoric acid biosynthesis. Data are mean ± SE for three biological replicates. Different letters above the bars indicate statistically significant differences at *p* < 0.05 for ANOVA. The expression levels of genes are presented using mRNA levels normalized to *ACT* [25].

As stated above, methyl jasmonate 7.5 mg/L (MeJa) was chosen as the optimal elicitor concentration to stimulate chicoric acid and other polyphenols biosynthesis in *E. purpurea* cell suspensions (Figure 1). To test the effects of MeJa on *E. purpurea* cell suspensions, first we evaluated the gene expression in the maintenance medium (T0-T1), then in the productive medium without MeJa (T2), and finally in the productive medium added with MeJa (T3-T4-T5) (Figure 4) at the same time-points studied to quantify the total content of polyphenols and chicoric acid (Figure 2). *PAL* expression showed the highest (4-times up-regulation if compared to T0) transcription at T2, so after 7 days in the productive medium but before the addition of MeJa, to decrease thereafter, reaching at T5 values 2-times higher with respect to that measured at T0 (Figure 4A). A similar trend was observed for *4CL*, with the highest rate in T2, and a rapid decrease in T3, remaining significantly invariant between T3 and T5 (Figure 4C). Nevertheless, the level of *4CL* expression was higher than *PAL* at T2, with an increase of almost 9-times if compared to T0, and of 5-times at T3-T4-T5. Conversely, *C4H* levels remained constant between T0 and T3, showing a significant downregulation only in T4 and T5 (−50%) (Figure 4B). A different trend was shown for *C3H*, which showed values slightly higher and similar to each other with respect to T0 at T1, T2 and T3 and a further significant increase in its expression at T4 and T5 (4-times up-regulation if compared to T0) (Figure 4D). *HTT* displayed two peaks at T2 and T4 of a 3.5-times up-regulation if compared to T0 but displayed a halving at T5 if compared to T4 (Figure 4E). *JAZ1*, involved in the jasmonate signaling, showed a constant increase from T1 to T4, reaching a 4-times up-regulation at T4 if compared to T0. However, at T5 its expression decreased of a half if compared to T4 (Figure 4F).

## 3. Discussion

*Echinacea purpurea* (L.) Moench is a perennial herbaceous plant that produces many bioactive compounds with pharmacological activities, such as alkylamides, polysaccharides, glycoproteins, flavonoids and phenolic compounds [26]. Among the phenolic compounds, there are the derivates of caffeic acid (CADs), with chicoric acid representing the most important CAD of *E. purpurea,* particularly abundant in the aerial parts and studied for its antioxidant, anti-inflammatory, antiviral and immunostimulatory properties [7].

Despite their huge employments in commercial products derived from *Echinacea*, CADs concentrations, and consequently the chicoric acid content, vary according to many endogenous and environmental factors. These lead to qualitative and quantitative inconsistencies in bioactive compounds containing *Echinacea*-derived products [21].

Plant cultures are an attractive alternative source for the production of high-value secondary metabolites, such as chicoric acid [27]. However, the productivity of secondary metabolites in plant culture is comparatively low; but it has been hypothesized that a targeted and accurate manipulation of culture medium could allow to obtain valuable secondary metabolites in large scale [28]. For instance, the application of elicitors is one of the most effective methods for enhancing the production of secondary metabolites. Accordingly, it was shown that a treatment with the phytohormone methyl jasmonate (MeJa) induced the accumulation of phenolics in *E. purpurea* cell cultures [22].

In this study, a liquid cell culture of *E. purpurea* was obtained and MeJa was utilized as an elicitor to induce the biosynthesis of total polyphenols and of chicoric acid. *E. purpurea* cell suspension was initially conducted in a liquid medium without any elicitors (maintenance medium), then sub-cultured in a productive medium to increase the production of total polyphenols and chicoric acid. The most appropriate MeJa concentration to boost the chicoric acid production was 7.5 mg/L (Figure 1). A smaller increase in the chicoric acid content was also noticed in the absence of MeJa after 7 days of cell culturing in the productive liquid medium, probably due to endogenous jasmonic acid biosynthesis and/or action. Furthermore, the productive medium appeared to induce the biosynthesis of chicoric acid equivalent (P-CAe) independently from MeJa provision, which in turn seemed to be more specifically effective at inducing chicoric acid biosynthesis.

We evaluated the transcription of *JAZ1* as a marker for JA-signaling in cell culture. JAZ proteins were found to be the substrates of SCF^COI1^, acting as negative regulators of the transcriptional regulator JIN1/MYC2 to suppress JA responses [29]. In response to increased JA levels, the JA receptor Coronatine Insensitive 1 (COI1) ubiquitinates JAZs, tagging them for degradation through the 26S proteasome, thereby releasing downstream transcription factors to regulate gene expression and activating JA-related responses [30]. In this study, the expression of *JAZ1* was up-regulated already in T2 (Figure 4F), before the addition of MeJA, thus supporting the hypothesis that endogenous JA signaling also participated in this pathway, as confirmed by the parallel induction of polyphenol synthesis (Figure 2B).

Nevertheless, the addition of MeJa between T2 and T3 further stimulated both the transcription of *JAZ1* and the accumulation of chicoric acid thatpeaked after 12 days in the production medium, 5 of which were in the presence of MeJa elicitation (T4, Figure 2). These results highlighted the relevance of *JAZ1* as a marker gene which can precociously detect the JA impact on metabolites production in *E. purpurea* cell cultures, both as an endogenous molecule and as an exogenous elicitor (MeJA).

The trend of transcription of some genes putatively involved in the chicoric acid biosynthesis in response to the time of permanence in maintenance and productive medium and to the provision of MeJa was also assessed (Figure 4).

Chicoric acid derives from p-coumaroyl-CoA, which is produced through the activities of four essential enzymes in the initial steps of the phenylpropanoid pathway (Figure 3): phenylalanine ammonia-lyase (PAL); cinnamate 4-hydroxylase (C4H), 4-coumarate: CoA ligase (4CL), and a bifunctional p-coumarate 3-hydroxylase/ascorbate peroxidase (C3H/APX) [31]. Finally, HTT (hydroxycinnamoyl-CoA: tartaric acid hydroxycinnamoyl transferase) catalyzes one of the final steps from caffeoyl CoA (Fu et al. 2021). In the present results, *PAL*, *4CL* and *HTT* (Figure 4A,C,E) showed an induction already at T2 in *E. purpurea* cell suspension. This suggested a trend similar to that observed for polyphenol accumulation, hence one independent from MeJa provision but dependent on the transfer to the productive medium. Accordingly, these three genes might be considered to respond to endogenous signals (e.g., jasmonate) as generic markers for polyphenols biosynthesis. Differently, *C3H* expression appeared correlated with the enhanced biosynthesis of chicoric acid in *E. purpurea* cell suspension and significantly boosted by MeJa elicitation, thus representing a promising marker for chicoric acid production (Figure 4D). Interestingly, *C3H* was the only gene among the five selected that showed markedly high levels of transcription in the ligulated florets with respect to the inflorescence (Figure S1), probably subtending a strong accumulation of flavonoids in the pink, sterile ray flowers [32].

C3H is the only reported hydroxylase involved in phenylpropanoid biosynthesis that is not a membrane-bound cytochrome P450. It can be considered as a metabolic deviation for phenolic ring 3-hydroxylation, directly from free p-coumaric acid to caffeic acid, the direct precursor of chicoric acid through the caffeoyl-CoA [33]. Moreover, C3H seems to play a crucial role in the reactive oxygen metabolism by its cytosolic ascorbate peroxidase activity that could oxidize both ascorbate and 4-coumarate [33]. Similar to CSE (caffeoyl shikimate esterase) that promote tolerance to cadmium-induced oxidative stress [34] and CCR (cinnamoyl CoA reductase) that acts as an effector of small GTPase Rac in the defense signaling in rice [35], C3H could be involved in stress responses. Actually, the production of polyphenols is strongly linked to the need to enhance the efficacy of the antioxidant system because of an altered redox homeostasis. According to this assumption, the relevance of C3H in the antioxidant cell response as a crucial component of both the non-enzymatic (polyphenols) and enzymatic (APX) response to oxidative stress is even more evident.

**Figure 5:**
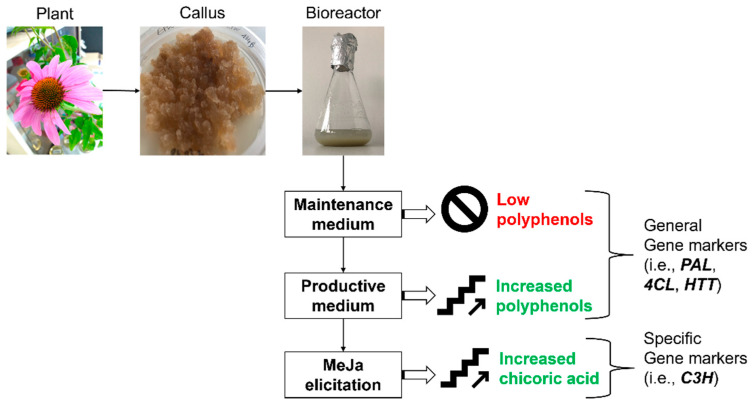
Summary diagram: the study provided a protocol to grow *E. purpurea* cell culture optimized for the production of caffeic acid derivatives and proposed *PAL* (*phenylalanine ammonia-lyase*), *4CL* (*4-hydroxyl cinnamoyl CoA ligase*), and *HTT* (*hydroxycinnamoyl-CoA: tartaric acid hydroxycinnamoyl transferase*) as generic markers for polyphenols biosynthesis, and *C3H* (p*-coumarate-3-hydroxylase*) as a specific marker for the enhanced biosynthesis of chicoric acid by methyl jasmonate (MeJa) elicitation.

In conclusion, this study provided an effective protocol to grow *E. purpurea* cell culture optimized to produce caffeic acid derivatives, in particular chicoric acid, and led to the identification of a suitable gene marker (*C3H*) useful for early predictions of the cell aptitude to synthesize these molecules. This enables a simpler, more reliable and more economically sustainable realization of the whole process (Figure 5).

## 4. Materials and Methods

### 4.1. Plant Material

For the callus induction, the *Echinacea purpurea* plant was bought from the nursery plant “Le georgiche” (Brescia, Italy). To test primer reliability and qPCR protocol, *E. purpurea* plants were directly purchased from “Isolaflor” (Ponte S. Nicolò, Padova, Italy).

### 4.2. Callus and Cell Suspension Culture Induction

Young tissues (shoots) from plants of *E. purpurea* were collected for the callus induction. Shoots were washed under running water and sterilized by means of a treatment in sequence with 70% (*v*/*v*) ethanol (Honeywell, Wunstorfer Straße 40, D-30926 Seelze, Germany) in water for about 15 s. They were then washed in 2% (*v*/*v*) sodium hypochlorite solution (6–14% active chlorine, (MERCK KGaA, 64,271 Darmstadt, Germany) and 0.1% (*v*/*v*) Tween 20 (Duchefa, Postbus 809, 2003 RV-Haarlem, The Netherlands) for about 5 min; finally, they were given at least 4 washes with sterile distilled water. The shoots were cut into small pieces (explants) of sub-centimetric dimensions (0.1–0.5 cm). The fragments of plant tissue were deposited in several Petri dishes containing solidified Gamborg B5 Medium [36] supplemented with 20 g/L sucrose (Sudzucker AG, Manheim, Germany), 1 mg/L of naphthalenacetic acid (NAA) (Duchefa), 0.5 mg/L of indolacetic acid (IAA) (Duchefa), 0.5 mg/L di Kinetin (K) (Duchefa) and 0.8% of plant agar (Duchefa), final pH 6.5 (*Echinacea purpurea*-medium, herein called EP-medium). Petri dishes containing explants were incubated at 25 ± 2 °C in the dark. Calli were grown after 6 weeks of incubation and were subjected to subculture for at least 6 months until they became friable and homogeneous, with a constant growth rate. The suspension cultures were obtained transferring a part of selected calli (10% *w*/*v*) in 1 L Erlenmeyer flasks containing 250 mL of liquid culture medium (EP-medium without plant agar). The suspension cultures were incubated at 25 ± 2 °C and placed in the dark on a rotary shaker at 120 rpm, for at least 7 days, to reach a fresh weight between 40–45% *v*/*v*. To induce the biosynthesis of chicoric acid and other polyphenols (chlorogenic acid, cynarine and caftaric acid), 10% (*v*/*v*) of the suspension culture from the liquid EP-medium (maintenance liquid medium) was transferred to 150 mL of final productive liquid medium (EP-F medium). The EP-F medium was a Gamborg B5 Medium supplemented with 40 g/L of sucrose, 1 mg/L of NAA, 0.5 mg/L of IAA, 0.5 mg/L of K, without plant agar and a final pH 6.5. The suspension culture in EP-F medium was incubated in the same conditions as the maintenance medium. After 7 days of fermentation in EP-F medium, the addition of 2.5, 5, 7.5 and 10 mg/L of methyl jasmonate (MeJa, Sigma) was evaluated to find the optimal concentration to induce the highest content of total polyphenols. Methyl jasmonate solution was prepared by dissolving 400 mg of MeJa in 10 mL of 70% (*v*/*v*) ethanol:water solution.

### 4.3. Cell Growth Time-Points

To evaluate the total polyphenol (caffeic acid derivatives, CADs’) content expressed as equivalent of chicoric acid (P-CAe) and the expression of CADs’ related genes during the cell suspension culture growth, several time-points were set (Table 1). At every time-point, 10 mL of cell suspension culture was sampled to analyze both P-CAe and chicoric acid content, while 50 mL, corresponding to 10–12 g of cells, was used to extract RNA and evaluate gene expression of target genes.

T0 and T1 are related to 5 and 7 days in the maintenance medium, respectively. After sampling the cells at T1, they were transferred to production medium and sampled after 7 days (T2). After this sampling, the elicitor MeJa was added. T3, T4 and T5 refer to 2, 5 and 7 days after MeJa supplement, respectively, corresponding to 9, 12 and 14 days after transfer to the production medium.

Three biological replicates were set for each time point.

### 4.4. Gene Bank Screening and Primer Design

Despite the diffuse use of *E. purpurea*, the complete genome sequence is still not available for this species. Nevertheless, three different studies obtained the RNA-seq dataset from the mature flower, primary stem, leaves (immature and mature) and whole plant of *E. purpurea* [4,24,37]. We used the unigenes annotated by Tahmasebi et al. [24] to find genes related to chicoric acid biosynthesis and to methyl jasmonate signaling. These sequences were used to design specific primers for quantitative Real-time PCR (qRT-PCR) using Primer3 v.0.4.0 (http://bioinfo.ut.ee/primer3-0.4.0/). The list of genes and primers used are reported in the Table S1.

### 4.5. RNA Extraction and cDNA Synthesis

To assess the reliability of the RNA extraction protocol, tissues from the *E. purpurea* plants were used as substrates to extract RNA. In a flowering plant, 3 tissues were sampled: the central yellow/brown florets, the pink ligulate florets, and young leaves. 100 mg of each tissue were sampled, in three independent biological repetitions, and immediately frozen in liquid nitrogen.

Regarding cell suspension cultures sampled at each time-point **(**Table 1), samples were centrifuged in 50 mL falcon tubes at 3000 rpm for 15 min, then the supernatant was discarded to isolate the cellular pellet. 50 mg of each cellular pellet were sampled, in three independent biological repetitions, and immediately frozen in liquid nitrogen.

Total RNA, from both tissue and cell culture, was extracted using SpectrumTM Plant Total RNA Kit (Sigma, St Louis, MO, USA). RNA was quantified with a Nanodrop1000 (Thermo Scientific, Nanodrop Products, Wilmington, DE, USA) and RNA quality was assessed via agarose gel electrophoresis. Some 500 ng of RNA for each sample were then reverse transcribed to cDNA as described by Manoli et al. [38].

### 4.6. Quantitative Real-Time PCR (RT-qPCR)

RT-qPCR was performed using the StepOne Real-Time PCR System (Applied Biosystems, Thermo Fisher Scientific, Waltham, MA USA), as described by Nonis et al. [39]. SYBR Green reagent (Applied Biosystems, Thermo Fisher Scientific, Waltham, MA, USA) was used in the reaction, according to the manufacturer’s instructions. Melting-curve analysis confirmed the absence of multiple products and primer dimers. Target gene relative expression was determined according to the Schmittgen and Livak method [40], using *ACTIN* as reference gene [25]. A list of putative housekeeping genes was also tested by means of Real-time PCR (Table S1), but *ACTIN* was found as the most appropriate housekeeping gene for the gene expression analysis. The results of gene expression were calculated according to the 2^−ΔCT^ method, normalized to a reference gene [40].

Three technical replicates were performed on three independent biological repetitions. ANOVA was performed as statistical analysis, with significance set to *p* < 0.05 using the web tool SATQPCR (http://satqpcr.sophia.inra.fr/cgi/home.cgi) [41].

### 4.7. UPLC-DAD Analysis of Chicoric Acid and Total Polyphenol Content

10 mL of *Echinacea purpurea* suspension culture were transferred into a 15 mL test tube, and 2 mL of a water solution containing 20 g/L of citric acid monohydrate (ACEF) and 10 g/L of ascorbic acid (Ph.Eur. E300-ACEF) were added for the quantification of the total polyphenols expressed as a chicoric acid equivalent. The suspension was mixed for 10 s and frozen at −20 °C until UPLC-DAD analysis. Before the analysis, the suspension was thawed and transferred into a 50 mL test tube and 10 mL ethanol were added. The suspension was mixed for 10 s with a vortex mixer and homogenized for 1 m with ultraturrax (T18 digital IKA) at 20,000 rpm in an ice bath; finally, it was centrifuged at 4500 rpm for 5 min at 6 °C. At the end of centrifugation, 2 mL of the supernatant were transferred into an Eppendorf test tube and further centrifuged at 14,000 rpm for 3 min at 6 °C. The supernatant was transferred into a new Eppendorf test tube and preserved in ice until loading into UPLC system. The sample was diluted in water and filtered over 0.22 μm filters before being loaded into the UPLC system. The chromatography system used for quantification of the total polyphenols expressed as chicoric acid equivalent (P-CAe) consists in an Acquity UPLC BEH C18 1.7 μm column, size 2.1 × 100 mm, coupled to an Acquity UPLC BEH C18 1.7 μm VanGuard Pre-Column 3/Pk, size 2.1 × 5 mm. The platform used for the UPLC-DAD analysis was comprised of a UPLC system (Waters Corporation, Milford, MA, USA) consisting of an eluent management module(Binary Solvent Manager model I Class) and of an auto-sampler (Sample Manager FTN model I Class) coupled to a PDA eλ diode array detector. Empower 3 (Waters) software was used to acquire and analyse the data. The chromatography method used was the following: solvent A: water, 0.1% formic acid; solvent B: 100% acetonitrile. The initial condition was 99% solvent A; moreover, the flow remained constant at 0.350 mL/min throughout the duration of the analysis. The chromatography column was temperature controlled at 30 °C. Elution of the molecules was conducted by alternating gradient and isocratic phases, as indicated in Table 2.

**Figure 6:**
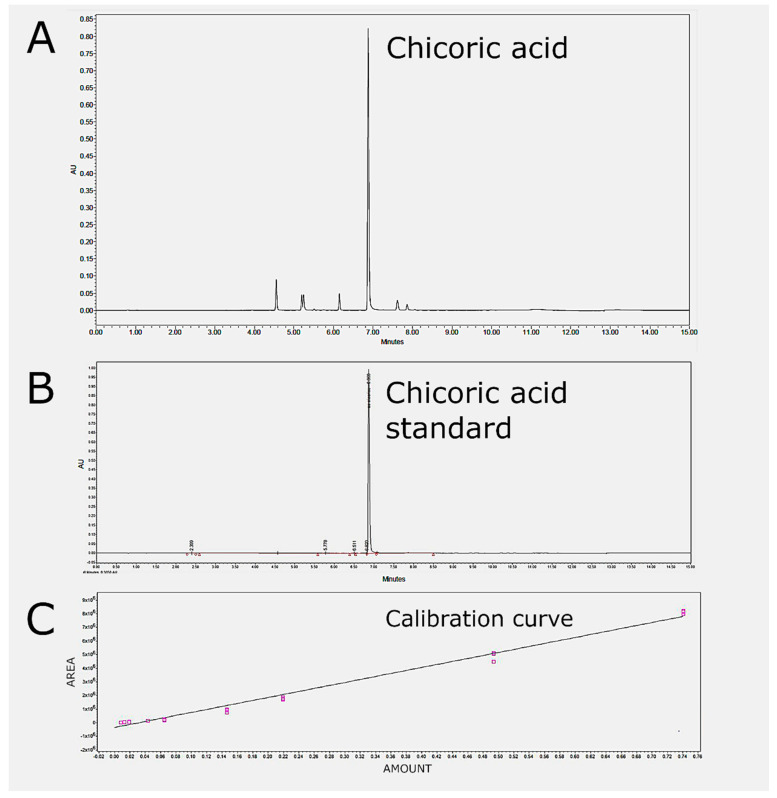
**Panel** (**A**): Chromatographic profile of the *Echinacea purpurea* suspension culture extract. The main peak at retention time 6.8 min corresponds to chicoric acid. **Panel** (**B**): Chromatographic profile of chicoric acid commercial standard. The retention time of chicoric acid peak is at 6.8 min. **Panel** (**C**): Calibration curve of chicoric acid; values are mean of three independent measures.

Quantification of total polyphenols and chicoric acid in the samples was based on absorbance of UV/VIS spectra, measured at 330 nm. The amount of chicoric acid and the P-CAe (total polyphenols content expressed as chicoric acid equivalent) were evaluated through the comparison with a calibration curve obtained from serial dilution of the commercial standard of chicoric acid (CA 6537-80-0; purity ≥ 95%; Sigma Adrich). The data analysis was carried out with Empower 3 software. P-CAe were quantified by comparing all the peak areas measured at 330 nm wavelength against the areas of the calibration curve of the reference standard chicoric acid. The chicoric acid content was quantified by comparing the peak area at retention time 6.8 min (Figure 6A) measured at 330 nm against the area of the calibration curve of the reference standard chicoric acid (Figure 6B).

Calibration curve was obtained by the external standard method, using nine different concentrations (0.148, 0.099, 0.044, 0.029, 0.013, 0.009, 0.004, 0.003 and 0.002 µg/µL) of the commercial standard of chicoric acid (CA 6537-80-0; purity ≥ 95%; Sigma Adrich) with three injections per amount (Figure 6C). The chicoric acid was initially solubilized in ethanol at a concentration of 1 µg/µL. Starting from this solution, three technical replicas of the serial dilution were carried out.

The peak areas measured at 330 nm wavelength were plotted against the known concentrations of the standard solutions to establish the calibration equation; a linear regression equation was calculated via the least-squares method. The calibration line was obtained by indicating on the abscissa the quantity, in μg, of each standard injected into the chromatographic column (Amount) and on the ordinate the value of the area underlying the analyte peak. The equation of the line, obtained by averaging the values of the three technical replicas was as follows: y = 1.10 × 10^7^X − 3.48 × 10^5^. The correlation coefficient (R2) of the regression equation presented was 0.994, as shown in Figure 6C.

## 5. Patents

Patent ITA102019000004119-PCT/IB2020/052591: phytocomplex and extract of a meristematic cell line selected from *Echinacea purpurea*.

## Figures and Tables

**Table 1:** Cell growth time-points chosen for sampling of *Echinacea purpurea* cell suspensions.

Time-Point	Growth Condition	Type of Nutrient Medium
**T0**	5 days in the maintenance medium (liquid EP-medium)	Maintenance medium (liquid EP-medium)
**T1**	7 days in the maintenance medium (liquid EP-medium)	Maintenance medium (liquid EP-medium)
**T2**	7 days in production medium (EP-F medium)	Production medium (EP-F medium)
**T3**	9 days in production medium (EP-F medium)	Production medium (EP-F medium) with MeJa for 2 d
**T4**	12 days in production medium (EP-F medium)	Production medium (EP-F medium) with MeJa for 5 d
**T5**	14 days in production medium (EP-F medium)	Production medium (EP-F medium) with MeJa for 7 d

Abbr: d, days; EP-medium, *Echinacea purpurea*-medium; EP-F medium, final productive liquid medium.

**Table 2:** Elution of the molecules in UPLC-DAD analysis.

Time from Start of the Analysis (Minutes)	Percentage of Solvent B	Slope
0	1%	
1	1%	Linear
11	40%	Linear
12	100%	Linear
13	100%	Linear
13.10	1%	Linear
15	1%	Linear

## Data Availability

All data generated or analyzed during this study are included in this published article.

## Supplementary Materials

The following supporting information can be downloaded at: https://www.mdpi.com/article/10.3390/ijms231911179/s1.
