# Supplementary material for: C3H Expression Is Crucial for Methyl Jasmonate Induction of Chicoric Acid Production by Echinacea purpurea (L.) Moench Cell Suspension Cultures
Source: Int J Mol Sci. 2022 Sep 23;23(19):11179. doi: 10.3390/ijms231911179 (PMC9570471; doi:10.3390/ijms231911179)
Supplement: Supplementary file 1 [file ijms-23-11179-s001.zip › Figure S1.pdf]

Article

C3H expression is crucial for methyl jasmonate induction of chicoric acid production by *Echinacea purpurea* (L.) Moench cell suspension cultures

Laura Ravazzolo[1], Benedetto Ruperti[1], Marco Frigo[2], Oriana Bertaiola[2], Giovanna Pressi[2], Mario Malagoli[1], Silvia Quaggiotti[1*]

[1] Department of Agronomy, Food, Natural resources, Animals and Environment, University of Padova, viale dell'università 16, 35020 Legnaro, PD, Italy

[2] Aethera Biotech srl, Via dell'Innovazione 1, 36043 Camisano Vicentino VI, Italy

* Correspondence: silvia.quaggiotti@unipd.it; Tel.: +39 049 8272913

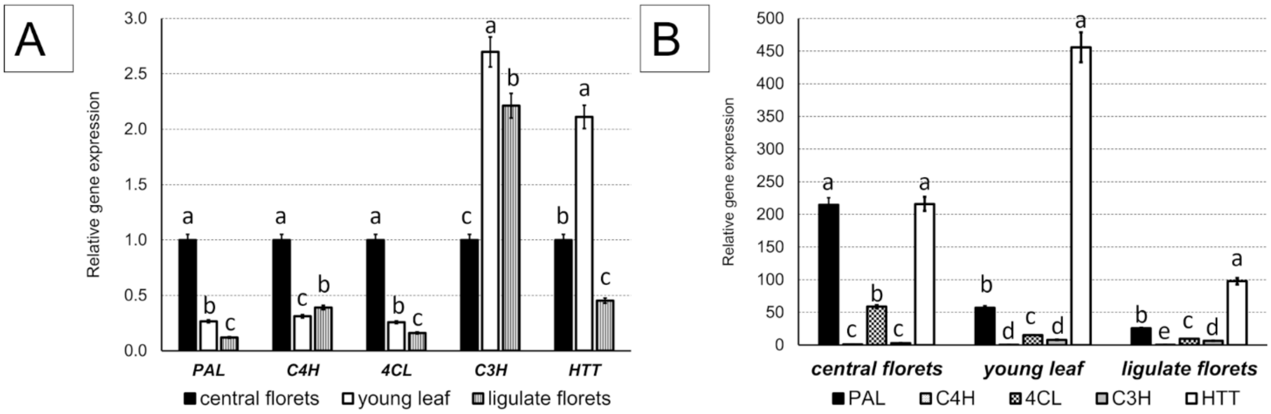

Figure S1. *In vivo* gene expression analysis of *Echinacea purpurea* genes involved in chicoric acid biosynthesis in different tissues. **Panel A** shows the relative gene expression of each gene with respect to the value in the central florets. **Panel B** shows the relative gene expression of every gene compared to the value of *C4H* in the central florets and expressed as 1. Data are mean ± SE for three biological replicates. The expression levels of genes are presented using mRNA levels normalized to *ACT*. Different letters above the bars indicate statistically significant difference at $p<0.05$ for ANOVA.

To set up the methodology, gene expression of genes related to chicoric acid biosynthesis was initially tested on samples obtained from *E. purpurea* tissues, in particular central florets, ligulate florets and young leaf (**Figure S1**). The expression of all the five genes representative of chicoric acid biosynthesis was markedly higher in central florets for *PAL*, *C4H* and *4CL*, while it was more than 2-times higher in the young leaf for *C3H* and *HTT*. The gene expression of all the chicoric acid biosynthesis gene appeared decisively down-regulated in the ligulate florets, with the only exception of *C3H* that showed a 2-times up-regulation in this tissue (**Figure S1A**).

Considering the level of expression in each tissue (**Figure S1B**), *PAL* and *HTT* were the most expressed in the central florets, with *HTT* showing the highest expression also in the young leaf and ligulate florets, followed by *PAL* and *4CL*. *C4H* and *C3H* appeared as the less expressed genes in the three tissues.
